# Editorial and Miscellaneous

**Published:** 1860-06

**Authors:** 


[From the Boston Medical Journal.]

In *The American Journal of the Medical Sciences* for April, 1858, Dr. Read published an article upon "the influence of the placenta on the development of the uterus during pregnancy." This was noticed by Brown-Sequard in the *Journal of Physiology* for January, 1859. This distinguished physiologist considers as entirely correct the view advocated by Dr. Read, viz., that the pait of the uterus which is first developed is that to which the placenta is attached, and adds, "to physiologists it is so clear that it must be so, that they do not understand how accoucheurs could have had any doubts with regard to the fact.

The same paper is referred to by Dr. J. M. Duncan, in the *Edinburgh Medical Journal* for March, 1859, in connection with some remarks upon placenta prævia. He says:

"On this subject I will not further enter, but refer to obstetric writers, and especially a recent American author, Mr. Read (*American Journal of the Medical Sciences*, April, 1858), for sufficient evidence of the extremely unsatisfactory nature of the notions now entertained on the subject. But, while Mr. Read has done good service in exposing the untenableness of the views now entertained, it is necessary to add that his own theory is, perhaps, the most untenable of all. For we find him supposing that the placenta may attach itself to the lowest part of the cervical portion—a supposition, I need scarcely say, quite inconsistent with all that is known of placenta prævia."

We were surprised to learn, a few days since, that the difference of opinion, expressed in a portion of the passage quoted, was entirely owing to a misunderstanding, long ago explained in a correspondence between the two writers.—The letter of Dr. Duncan we are now allowed to publish, and do so with the greatest pleasure, regretting exceedingly that it should have been so long withheld.

Edinburgh, July 9, 1859.

Dear Sir—I have just received your interesting note, and thank you sincerely for it. I am sorry that I should have misunderstood your meaning, when you spoke of the lowest part of the cervical portion; but, as you admit, the fault was not mine, but yours. This does not diminish my regret, for, apart from my desire to do full justice to your valuable paper, I have missed the value and interest of your researches, from believing them greatly vitiated by an error, which you now show me is a mere misinterpretation, arising from indistinctness of language.

I now see that when you spoke of the lowest part of the cervical portion, you meant the lowest part of the cervical end of portion of the body of the uterus. I thought your words indicated the part adjoining the external os uteri, while they were really intended to indicate the part of the cavity of the body adjoining the internal os uteri.

Perhaps you are not aware that it has been thought by some authors that in individual cases, and very rare ones, the placenta was really attached to the internal surface of the cavity of the cervix. But this is far from proved, and would require much proof to ensure acceptance; and, even if quite proved, it is so rare as not to affect the argument in papers such as yours and mine, which take up the common phenomena of placenta prævia.

I shall read your paper again, and with more intelligence now. Only, I must tell you that you have found meanings in my article that were never expressed in it, when you suppose me to believe the general scope of your article to be wrong. I am, on the contrary, inclined to adopt it, and always was so. Only, seeing the special part of your paper referring to placenta prævia to contain an error (now corrected), I held and said that that error vitiated your argument on this special point.

These explanations bring us, I think, to one mind; and I will venture to express a hope that we may meet again, if not in the body at least in literature, and then our present correspondence will enable us more justly to appreciate one another.

Dr. Read. Yours, most truly, J. Matthews Duncan.

